# A systematic review of work-related musculoskeletal disorders and risk factors among computer users

**DOI:** 10.1016/j.heliyon.2024.e25075

**Published:** 2024-01-22

**Authors:** Biruk Demissie, Eniyew Tegegne Bayih, Alelign Alemu Demmelash

**Affiliations:** a Department of Environmental Health, College of Health Science, Debre Tabor University, Debre Tabor, Ethiopia; b Department of Environmental Health, College of Health Science, Debre Markos University, Debre Markos, Ethiopia; c Department of Environmental Health and Hygiene, Bonn Universiy Hospital, Bonn, Germany

**Keywords:** Work-related musculoskeletal disorder, Musculoskeletal disorder, Prevalence, Body area, Computer user, Office worker, Risk factors

## Abstract

**Introduction:**

Rapid technological developments, especially in the use of electronic devices, have affected workers. MSDs are a major burden for both employees and employers, and in contemporary society. Millions of computer workers suffer musculoskeletal diseases and it is the most common cause of occupational illness in the USA and result in medical costs and absenteeism that cost the sector between $45 and $54 billion annually. A single review was done about WMSDs, however it only investigated neck and upper extremities disorders. Therefore, this study aimed to review epidemiological evidence about the prevalence and risk factors of overall WMSDs among computer user bankers and office workers.

**Methods:**

An extensive literature search was undertaken in the PubMed, Web of Sciences, Google Scholar, and Scopus databases. Articles published in peer-reviewed English-language journals were considered for inclusion criteria. Articles published in conference proceedings, reports, abstracts, and not full text were excluded. After a thorough search of databases, a total of 90 articles were discovered, and finally, 25 of them met the inclusion criteria and were investigated in detail.

**Result:**

The magnitude of WMSDs ranged from 33.8 to 95.3 %. The lower back, neck, upper back, and shoulder were the most affected body parts, and the elbow, hip/thigh, knee, wrist/hand, and ankle/feet were the least affected body parts. Prolonged computer use, being older, repetitive moments, female sex, working in awkward posture, low educational status, a lack of physical exercise, and ergonomics training were significantly affected by MSDs.

**Conclusion:**

and recommendation: The prevalence of WMSDS was very high, and as a result, an interventional study should be conducted. Reducing prolonged use of computers and working in the right posture will reduce the magnitude of MSD among computer users.

## 1 Introduction

Work-related musculoskeletal disorders (WMSDs) can be defined as impairments of bodily structures such as muscles, joints, tendons, ligaments, nerves, bones, and the localized blood circulation system, caused or aggravated primarily by the nature of the work itself or by the workplace environment [[1], [2], [3]]. MSDs are a major burden for both employees and employers, and in contemporary society, musculoskeletal problems are the most prevalent sort of work-related health issue [4]. Every year over 2.34 million people die at work from an occupational injury or disease. The distribution of work-related fatalities accounted in Europe 11.7 %, Oceania 0.6 %, Africa 11.8 %, America 10.9 %, and Asia 65.0 % [1]. MSDs decreases productivity at work due to sick leave, absenteeism, and early retirement, and is also costly in terms of treatment and individual suffering [5].

Rapid technological developments, especially in the use of electronic devices, have affected workers [6]. Modern office work has shifted the nature of occupations from being active to sedentary. Computer overuse has been linked to increasing WMSDs among office workers. One of the reasons for this concern is the transition from paper to computer work [7]. Computer workers are occupations that can potentially be affected by WMSDs [8,9]. Millions of computer workers suffer musculoskeletal diseases, which are the most common cause of occupational illness in the USA and result in medical costs and absenteeism that cost the sector between $45 and $54 billion annually [10].

The annual prevalence of WMSDs among computer users varied from 33.8 to 95.3 % [11,12]. The musculoskeletal health of workers is believed to be influenced by a variety of factors, including individual, organizational, psychological, and psychosocial aspects [13].

Physical exposures associated with repetitive jobs, static posture for the arms and neck, and office design [14,15], and psychosocial factors related to job characteristics, high quantitative job demands, having little influence on one's work situation, and limited support from coworkers or supervisors [[16], [17], [18]]. Being female, working in awkward working postures, work - related stress, prolonged computer use, repetitive motion, having longer work experience, older age, lack of physical activity, lack of ergonomics training, poor educational status, smoking, and alcohol consumption are significantly associated with WMSDs [1,4,9,[19], [20], [21], [22], [23], [24]]. Even though a review on the prevalence of WMSDs among computer users was published, it only investigated neck and upper extremity disorders. Therefore, this study aimed to review epidemiological evidence about the prevalence and risk factors of overall WMSDs among computer users. The results of this review will provide a meticulous summary of all available articles about WMSDs among computer users for academics and policymakers.

## 2 Methods

### 2.1 Inclusion and exclusion criteria

Only primary studies that examined the prevalence of WMSD and its associated factors among bankers and office workers were included. Articles published in peer-reviewed English-language journals were considered for inclusion criteria. Reports, abstracts, conference proceedings, and articles that were not published in full text were excluded.

### 2.2 Data extraction

The title and abstract of the studies were screened based on the preset criteria. Retrieved articles were evaluated based on their title, objectives, and methodology. Irrelevant and duplicate articles were removed, and the full text of the remaining articles that met the preset criteria was reviewed for inclusion. To extract the data, a form was prepared that contains: author names, year of publication, country, population, sample size, mean age, percentage of female gender, recall period, prevalence of MSD, risk factors, and response rate. Two separate researchers carried out the extraction (BD and ETB). They had a comprehensive conversation about any disagreements they had, and if they persisted, they consulted the third author (AAD).

### 2.3 Operational definitions of variables

Work-related musculoskeletal disorder is perceived pain, ache or discomfort for at least 2–3 workdays in last 12 months in any part of body region (neck, shoulder, upper back, lower back, hip/thigh, knee/leg, and ankle/foot and wrist/hand) caused by workplace exposures [1,20,21].

### 2.4 Search methods

An extensive literature search was undertaken in PubMed, Google scholar, Web of Sciences, and Scopus databases. Keywords used for the search were; “musculoskeletal disorders” OR “work-related musculoskeletal discomfort” AND “body area” AND “computer user” AND “bankers AND office workers.

### 2.5 Search results

**Fig. 1:**
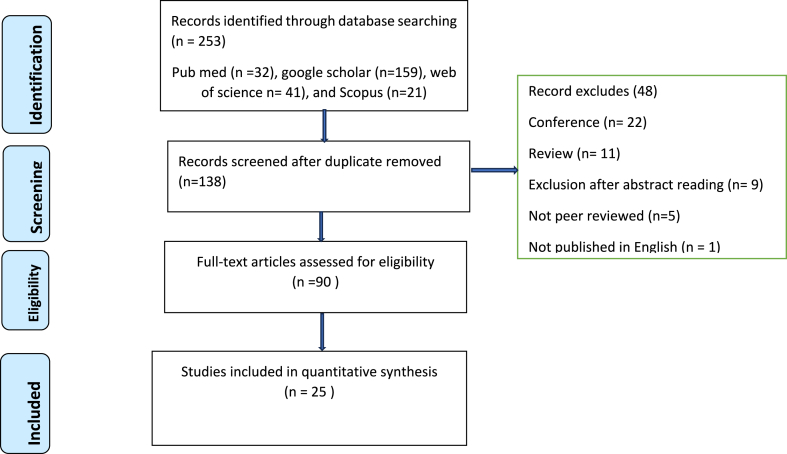
PRISMA flow diagram on prevalence of WMSDs and risk factors among computer users, 2023.

A total of 90 articles were discovered after a thorough search of the databases. The relevancy of the titles, keywords, and abstracts was checked, and this helped to weed out duplicate entries. A total of 25 potentially pertinent publications were obtained, and 65 papers were removed from the review after further examination of these publications because they failed to evaluate the prevalence of MSDs or present potential risk factors among computer users. The review also did not include any articles that did not detail a research study or literature review. The search process is shown in Fig. 1.

## 3 Results

### 3.1 Description of studies

A total of 253 articles were identified in the databases until February 28, 2023. 138 studies screened after duplicate or irrelevant articles were removed. Of 138 articles, 90 articles were fully evaluated. Finally, twenty papers satisfied the inclusion criteria and included in the final analysis. All studies were done using cross sectional study design. The sample size varied from 50 in Malaysia [7] to 838 Addis Ababa [1]. The subjects of all studies were computer users who were employed by various organizations. All the studies were conducted between 1996 in Hong Kong, China [25] and 2023 in Isfahan, Iran [26]. A total of 8883 respondents were participated from 14 different countries. Five studies were conducted from India [11,24,[27], [28], [29]], four from Ethiopia [1,8,20,21], three from Iran [26,30,31], two from China [25,32], two from Malaysia [7,33], and one study each from Bangladesh [4], Egypt [9], Saudi Arabia [34], Ghana [23], Pakistan [35], Kuwait [6], Nigeria [36], Sri Lanka [37], and Estonia [38]. The prevalence of WMSDs was obtained from all studies [1,4,[6], [7], [8], [9],^11^,^20^,^21^,[23], [24], [25], [26], [27], [28], [29], [30], [31], [32], [33], [34], [35], [36], [37], [38]], while the data regarding to associated factors were also reported by all except two studies [27,34].

### 3.2 Quality appraisal of the included studies

Two independent reviewers (BD and ETB) evaluated the quality of the included studies, and assigned a scored for the validity of results. The quality of each study was assessed using the Joanna Briggs Institute (JBI) quality appraisal criteria [39]. All studies were appraised using JBI checklist for cross-sectional study. Thus, among the twenty-five cross-sectional studies, eleven studies scored seven of eight questions, 87.5 % (low risk), nine studies scored six of eight questions, 75 % (low risk), and the remaining five studies also scored five of eight questions, 62.5 % (low risk). Studies with a quality evaluation indicator score of 50 % or above were considered low risk. After conducting a thorough quality appraisal, we concluded that the primary studies that were part of our analysis showed a high degree of reliability in their methodological quality scores. All studies scored between 5 and 7 out of a total of 8 points. Therefore, all included studies had good quality (supplemental file 3).

### 3.3 Risk of bias assessment

The risk of bias was evaluated using the risk of bias assessment tool [40]. It consists of ten items that assess four areas of bias: internal validity and external validity. Items 1–4 evaluate selection bias, non-response bias, and external validity. Items 5–10 assess measure bias, analysis-related bias, and internal validity. As a result, out of twenty-five included studies, fourteen studies scored eight or above of ten questions, five studies scored seven of ten questions, and six studies scored six of ten questions. Studies were categorized as ʺlow riskʺ if eight or above of ten questions answered a ʺYesʺ, as ʺmoderate riskʺ if six to seven of ten questions answered a ʺYesʺ, and as ʺhigh riskʺ if five or lower of ten questions answered a ʺYesʺ. Therefore, all included studies had low risk of bias (supplemental file 3).

### 3.4 Prevalence of MSDs

From 1996 until 2023, a total of twenty-five studies were reviewed. The prevalence of MSDs was highest (95.3 %) and lowest (33.8 %) in Shah Alam, Malaysia [33] and Tamil Nadu, India [11] respectively as shown in Table 1. According to Table 2, fifteen out of twenty-five studies reported that the lower back, neck, upper back, and shoulder were the most affected body parts, and the elbow, hip/thigh, knee, wrist/hand, and ankle/feet were the least affected body parts, respectively [1,4,[6], [7], [8],^11^,[20], [21], [22], [23],^25^,^28^,^30^,^31^,^34^].

**Table 1:** Study characteristics with the summarized prevalence of WMSDs and risk factors among computer user, 2023.

Author name (year, country)	Population	Sample size	Mean age in year	Sex (female in %)	Recall period (month)	Prevalence of WMSDs (%)	Risk factors	Response rate (%)
Dagne et al. (2016/17, Addis Ababa, Ethiopia	Bank worker	838	29.4 ± 5.91	50.7	12	77.6	Being female, awkward posture, no work time break, fixed position, type of chairs, and job stress	90
Dixit et al. (2019, Mangaluru, India)	Bank worker	487	39.96 ± 11.76	41.7	12	57.3	NS	65.5
Amin et al. (2015, Dhaka, Bangladeshi)	Bank worker	400	33.58 ± 12.326	30.8	12	77.8	Being older age, female sex and duration of computer use	100
Noha S. Elshaer (2016, Alexandria, Egypt)	Bank and telecommunication worker	258	NS	54.97	12	54.1	Female sex, longer working experience, working in awkward posture, inappropriate office equipment, task complexity break autonomy, low decision authority and low break quality	81.8
Darvishi et al. (2013, Kurdistan, Iran)	Bank worker	240	36.28 ± 3.87	22	12	78.5	Being older age, female sex, longer working experience and work load	83.3
Pate et al. (2018/19, Bhopal, India)	Bank worker	272	34.8 ± 10.6	32.05	12	41.1	Being female sex	95.2
Abdullah S. and Abdullah M (2020/21, Buraydah, Saudi Ariba)	Bank worker	397	33.8 ± 5.9	0	12	89.7	NS	44
Jubilant K and Godfred K (2012, Kumasi, Ghana)	Bank worker	400	30.6 ± 5.4	51.7	12	83.5	Being female sex, job tenure and high GHQ score	57.5
Khan et al. (2017/28, Hayat, Pakistan)	Bank worker	110	35 ± 8.0	17	12	71.8	Working awkward posture, working the same position for long period, repetitive movement, short rest time and no ergonomics training	100
I. T. S. Yu & T. W. Wong (1996, Hong Kong, China)	Bank worker	151	26.5	42.1	12	20	Longer work experience, longer working hour, working awkward posture, repetitive movement and work station design	80
Dixit et al. (2014, Punjab, India)	Bank worker	60	NS	NS	12	83.5	Older age, being male sex, smoking, drinking, working awkward posture and working longer duration	71
Sulaiman er al (2014/15, Tamil Nadu, India)	Bank worker	300	33.6 ± 10.5	40.2	12	33.8	Female sex, psychosocial stress & job tenure	54.7
Etana et al. (2019, Jimma, Ethiopia	Bank worker	335	31 ± 5.27	23.9	12	73.1	Having more working experience, working in awkward posture, job stress, alcohol consumption & working in same position	98
Akrouf et al. (2006, Kuwait)	Bank worker	800	33.2 ± 9.1	52.5	12	80	Female sex & having high GHQ score	93.8
Demissie et al. (2021, South Gondar, Ethiopia)	Bank worker	422	29.2 ± 9.1.	26.8	12	58.8	Older age, luck of physical exercise, luck ergonomics training and working in awkward posture	100
Kibret et al. (2018, Mekelle, Ethiopia)	Bank worker	338	29 ± 5	35.5	12	65.5	Older age, having high work experience, low educational status, lack of physical exercise, luck of ergonomics training, working awkward posture, having less break time and job stress	93.6
Maduagwu et al. (2013/14, Maiduguri, Nigeria)	Bank worker	350	31.54 ± 8.71	23.45	12	71.68	Work load, luck of breaks & ergonomics training & working in the same position	78.5
Ranasinghe P et al. (2009, Colombo, Sri Lankan)	Telecommunication worker	450	38.2 ± 9.5	57.3	12	63.6	Work load, luck of breaks, working in awkward posture,	97.7
Shanshan Wu et al. (2008, China)	office worker	720	31.6 ± 7.9	42.3	12	34.1	Female sex, Computer working hours, working in awkward posture, having no break, female sex, low educational status & no physical exercise	86.3
Oha K et al. (2008, Estonian)	University office worker	415	40.0 ± 10.0	85	12	77	Female sex, older age, low job security, emotional exhaustion and low job support	53
Latha S et al. (2011, Karnataka, India)	Office worker	783	37 ± 6.8	31.1	6	57.7	Female sex & being junior employees	92.3
Navidi F et al. (2021, Qazvin, Iran)	Office worker	101	39.26 ± 5.56	52.6	12	52.6	Working in awkward posture, work place layout, ergonomics training and physical exercise	19
Aziz A & Azmi N (2022, Malaysia)	University office worker	50	NS	60	12	44.2	Prolonged computer use and prolonged sitting	100
Habibi E et al. (2022, Isfahan, Iran)	University office worker	96	35 ± 9.4	45.8	12	50	Having longer work experience, female sex & older age	100
Noraziera Z & Norzaida A(2018, Shah Alam, Malaysia)	Office worker	110	NS	48.8	12	95.3	BMI & light physical exercise	78.2

NS: not specified, GHQ score: General health question.

**Table 2:** the prevalence of WMSDs in different body regions among computer users, 2023.

Author (year, country)	MSDs prevalence by body region (%)								
	Lower back	Upper back	Neck	Shoulder	Elbow	Wrist/hand	Hip/thigh	Knee	Ankle/foot
Dagne et al. (2016/17, Addis Ababa, Ethiopia	54	35.4	38	40.9	13.3	16.6	18.9	21.3	15.1
Dixit et al. (2019, Mangaluru, India)	27.1	14.2	26.9	20.7	7.5	15.2	6.4	12.5	22.7
Amin et al. (2015, Dhaka, Bangladeshi)	48	22	75.2	30.8	8.5	19	6	10.8	5.2
Darvishi et al. (2013, Kurdistan, Iran)	44	36	48	26	12	20	8	12	20
Noha S. Elshaer (2016, Alexandria, Egypt)	NS	NS	69	70	NS	73	NS	NS	NS
Pate et al. (2018/19, Bhopal, India)	34.7	52.2	55.2	49.8	12.3	24.3	12.7	11.2	6.5
Abdullah S. and Abdullah M (2020/21, Buraydah, Saudi Ariba)	66.9	41.7	59.4	51.4	16	26.9	26.3	33.7	43.4
Jubilant K and Godfred K (2012, Kumasi, Ghana)	64.8	61.7	47.4	37.4	15.2	3.4	13.9	2.2	6.5
Khan et al. (2017/28, Hayat, Pakistan)	18.5	NS	14.8	12.1	2.5	NS	3	3	2.5
Dixit et al. (2014, Punjab, India)	40.4	39.5	38.6	15.2	10	36.8	6.2	2.2	36
Sulaiman er al (2014/15, Tamil Nadu, India)	51.8	39.6	48.2	40.2	5	6	3	2	4
I. T. S. Yu & T. W. Wong (1996, Hong Kong, China)	NS	NS	31.4	16.5	NS	14.9	NS	NS	NS
Etana et al. (2019, Jimma, Ethiopia	54	42.7	45.4	37.9	9	14	8.4	13.7	11.3
Akrouf et al. (2006, Kuwait)	51.1	38.4	53.5	49.2	11.5	28.3	13.3	22.9	16.8
Demissie et al. (2021, South Gondar, Ethiopia)	38.9	32.9	45.3	26.4	16.3	19.7	12.8	15.2	10.6
Kibret et al. (2018, Mekelle, Ethiopia)	40.4	33.6	35.2	29.6	18.2	15.3	10.4	16	11.1
Maduagwu et al. (2013/14, Maiduguri, Nigeria)	45.1	32.3	56.6	46	15.9	17.2	14.2	19.9	14.6
Shanshan Wu et al. (2008, China)	6.6	26.2	55.5	50.7	NS	31.5	NS	NS	NS
Ranasinghe P et al. (2009, Colombo, Sri Lankan)	NS	NS	37.1	34.3	11	21.4	NS	NS	NS
Navidi F et al. (2021, Qazvin, Iran)	63.2	31.6	52.6	42.1	15.8	26.4	31.6	42.1	10.5
Oha K et al. (2008, Estonian)	42	NS	51	30	NS	35	NS	NS	NS
Aziz A & Azmi N (2022, Malaysia)	62	64	78	72	NS	NS	NS	NS	NS
Noraziera Z & Norzaida A (2018, Shah Alam, Malaysia)	70.9	44.2	65.1	68.6	20.9	50	25.6	4.9	33.7
Habibi E et al. (2022, Isfahan, Iran)	NS	NS	54.9	NS	43.2	31.5	NS	39.6	18.9

NS: not specified.

According to a study conducted in Estonia, pain was most frequently reported in the neck and lower back, while shoulder and wrist/hand pain were less common [38]. While a Qazvin, Iran study found the lower back, neck, knee, shoulder, upper back, and hip/thigh were the most common sites of pain, the elbow, wrist/hand, and ankle/foot were reported less often [30]. A Shah Alam, Malaysia [33] study found that the lower back, shoulder, and neck were the most affected body parts, while the ankle/feet, hip/thigh, and elbow were the least affected. A similar study done in Isfahan, Iran [26] indicated that the neck, back, and elbow were the most frequently affected body parts, whereas the hand/wrist, knee, foot/ankle were less affected. A China study reported that the neck and shoulder were the body areas most often impacted, while in contrast to other studies, the lower and upper back were the least affected body parts [41] as presented in Table 2.

### 3.5 Neck and shoulder pain/discomfort

10 of the 25 articles discovered that neck pain was the most prevalent MSDs [4,[6], [7], [8],^22^,^25^,^26^,^31^,^38^,^41^] and the highest and lowest prevalence of neck pain was observed in Malaysia (78 %) [7] and Hayat, Pakistan (14.8 %) [42], while the highest and lowest prevalence of shoulder pain was found in Malaysia (72 %) [7] and Hayat, Pakistan(12.1 %) [42] respectively. In the majority of studies that were reviewed, along with the neck, the lower back, upper back, and shoulder were listed as the body parts that were most affected [1,4,[6], [7], [8],^11^,[20], [21], [22], [23],^25^,^28^,^30^,^31^,^33^,^34^,^38^,^41^] as shown in Table 2.

### 3.6 Lower and upper back pain/discomfort

The prevalence of lower back pain has been presented in Tables 2 and it varies from 6.6 % in China [41] to 70.9 % in Shah Alam, Malaysia [33]. According to a study report, lower back pain was one of the leading MSDs among computer user employees. Among 25 studies, 12 articles reported that lower back pain was the most prevalent MSD throughout the body [1,11,[19], [20], [21],^23^,^24^,^28^,^30^,^33^,^34^,^42^]. Upper back pain was also one of the top-listed MSDs next to lower back and neck pain [1,4,[6], [7], [8],^11^,[20], [21], [22], [23],^25^,^28^,^30^,^31^,^34^]. The highest prevalence of upper back pain was reported from Malaysia (64 %) [7], and the lowest was from Mangaluru, India (14.2 %) [19]. However, contrary to other studies, a China study reported that the lower back and upper back were the least affected body parts by MSDs [41] as presented in Table 2.

### 3.7 Upper limb (wrist/hand and elbow) pain/discomfort

Most studies confirmed that computer users' wrists/hands, and elbows were the least affected bodily parts, followed by hip/thigh, knee, and ankle/foot [1,4,[6], [7], [8],^11^,[20], [21], [22], [23],^25^,^28^,^30^,^31^,^34^] (Table 2). The highest prevalence of wrist/hand and elbow disorders was recorded in Alexandria, Egypt [9], and lowest in Isfahan, Iran [26]. The prevalence of wrist/hand and elbow was ranged from 2.5 % to 73 % and 3.4 %–43.2 %, respectively.

### 3.8 Lower limb (hip/thigh, knee and ankle) pain/discomfort

According to Table 2 report, hip/thigh, knee, and ankle were reported as the least affected body parts. The lowest prevalence of hip/thigh, knee and ankle/foot were reported in Tamil Nadu, India (3 %) [11], Tamil Nadu, India (2 %) [11] and Hayat, Pakistan (2.5 %) [35] respectively, while the highest were 31.6 % in Qalvin, Iran [30], 42.1 % Qalvin, Iran [30] and 43 % in Buraydh, Saudi Arabia [34] respectively (Table 2).

## 4 Risk factors

Prolonged computer use [4,9,23,41], having older age [8,21,24,26,38] repetitive movement [25,42], having short rest periods [21,37,42], stress [1,11,20], having a high GHQ score [6,9,23], working in the same position for a long period of time [19,20,42], having heavy work load [31,37], smoking [24], extended work experience [20], lack of regular physical exercise and ergonomics training [8,21,33,41], poor educational status [41] and BMI(33) were significantly associated with MSDs as reported in Table 1.

## 5 Discussion

To determine the prevalence and risk factors of WMSDs in different body regions, 24 of the 25 studies used the standardized Nordic Questionnaire, while one study from Alexandria, Egypt, used the musculoskeletal upper extremity questionnaire.

The highest prevalence of MSDs was reported in Shah Alam, Malaysia (95.3 %) [33], while the lowest was in Tamil Nadu, India (33.8 %) [11]. This discrepancy might be due sample size variability between the two studies. The smaller sample size may lead to the highest prevalence of MSDs.

Twelve of the total reviewed articles reported that the lower back was mentioned as the most frequently affected body part [1,11,[19], [20], [21],^23^,^24^,^28^,^30^,^33^,^34^,^42^], whereas ten out of the total articles stated that neck pain was the most prevalent MSD [4,[6], [7], [8],^22^,^25^,^26^,^31^,^38^,^41^]. When using a computer for an extended period of time, the neck and thoracic extensor muscles must contract in order to keep the head positioned forward. This could lead to different stress zones and postural neck pain [7]. Tilting the neck more than 30° greatly increases the rate of fatigue in the neck muscles [43]. The head is supported by the neck; when the neck is bent, the head partially loses the support of the spine. The small spinal muscles therefore exert twice as much effort as usual to maintain the bent posture of the head. To maintain head stability in this posture, neck muscles must exert a lot of force. As a result, they become overwhelmed and eventually injured [44].

In most studies, the lower back, neck, upper back, and shoulder body parts were the most affected [1,4,[6], [7], [8],^11^,[20], [21], [22], [23],^25^,^28^,^31^,^34^], and the elbow, hip/thigh, knee, hand/wrist, and ankle/feet were the least affected [6,8,[20], [21], [22], [23],^34^]. According to studies conducted in Shah, Malaysia, and Hayat, Pakistan, the lower back, neck, and shoulders were the most severely affected bodily parts [7,33,42]. Another study found in Punja, India, revealed that the body parts with the greatest impact were the lower back, upper back, neck, wrist/hand, and ankle/feet, and the least impacted were the knee, hip, thigh, elbow, and shoulder [24]. Similar studies done in Estonia [38] and Quzvin, Iran [30] stated the neck, lower back, wrist/hand, and shoulder, as well as the lower back, neck, knee, shoulder, upper back, and thigh, were the most affected body areas, respectively. In addition, a study in Isfahan, Iran, found that the elbow was one of the most affected body parts [26], and a study in China reported that the neck and shoulder were the most affected body parts, and in contrast to many other studies, the lower back and upper back were the least affected [41].

Many findings show that female respondents were more likely to develop MSDs than males [1,6,9,11,22,23,26,41]. This is probably due to the fact that women are more often exposed to physical and psychosocial risk factors [45], and they have weaker muscles and shorter statures than men [46]. Women may experience a higher relative musculoskeletal load when performing the same activities as men because most workplaces are designed using anthropometric data from males [[47], [48], [49]], and they may also have a dual role as both workers and housewives (household work and childcare [25]). Repeated activity, long-term involvement in static work, inadequate rest, awkward postures during work with computers [26], small size body and low muscle mass are common causes of more MSDs in women than men [50,51]. Another possible explanation could be differences in exposures to work-related factors and vulnerability [23,52,53], hormonal differences, psychosocial and cultural phenomena have been documented to play some roles [54] The ‘stress’ was associated with the emergence of pain in some parts of the body in men, whereas the ‘job dissatisfaction’ factor was more strongly correlated with WRMD symptoms in women [55].

However, research conducted in Punjab, India, found that men were experienced more MSDs than women [24]. This might be because men are more likely to smoke and drink alcohol. As a result, this probably leads to a higher prevalence of MSDs among men than women.

**Having older computer users, respondents** were significantly affected by MSDs [8,21,24,26,38]. This could be as a result of older people having more exposure to various risk factors than younger people [8]. The pathogenesis of musculoskeletal diseases has been connected to biological changes brought on by aging, such as degenerative changes to muscles, tendons, ligaments, and joints [56]. Another explanation could be that as people get older, they gain more work experience, which in turn leads to increased fatigue and muscle tension over a long period of time, eventually leading to WMSDs [21]. As age increases, the level of physical activity decreases which predisposes individuals to obesity [19].

However, Nigerian research found that the age group of 20–29 years had a higher prevalence of WMSDs than the oldest age group (40 years and older) [22]. This could be the lowest age group and have lower professional experience, knowledge, and skills. Increased work load could be another contributing factor to the higher prevalence of WMSDs compared to other age groups [22]. As their ranks rise, they transition from direct banking jobs to administrative roles, which require less physical demanding [22].

Respondents with high working experience were significantly affected by MSDs [7,20,21,23,26]. With more work experience comes a higher chance of developing WMSDs, and the likely cause raises tiredness and muscular tension for years [7]. People with more experience increase their exposure to risk factors as compared to those with less experience, and WMSDs are cumulative traumas or repetitive strains that develop gradually as a result of overuse [20].

Working in awkward posture (sitting with their back twisted or bent) was more likely to develop WMSDs than sitting with their back straight [1,8,9,20,21,24,25,30,37,41,42]. This is because bad posture can cause stiffness and compression over all skeletal and muscular areas, resulting in pain and discomfort throughout the body [2,41]. Muscles and joints are involved in an activity, and the amount of force generated is determined by the body posture because, as the backbends, twisting or bending of the shoulders, wrists, hips, and knees can increase the stress on the joints, muscles, and nerves and cause fatigue, leading to injuries [20].

WMSDs are more likely to develop when working in a fixed posture than when working in an unfixed position [1,7,20,42]. According to studies done in Adis Abeba [2] and Jimma [12], respondents who held fixed positions had a 1.8 and 2-fold higher chance of developing WMSDs than those who did not, respectively. This could happen when the muscles are operating in a fixed position without having a chance to relax, which reduces blood flow [57], and our body is not designed to remain static but to move about [20]. Furthermore, continuing to work in the same posture for an extended period of time increases the muscular load and activity around the facet joint, which causes joint compression and affects the symptoms of the musculoskeletal system [58].

Respondents working with stress had a higher risk of developing WMSDs compared to non-stressed respondents [1,6,11,20,21,23]. Studies done in Addis Ababa [1], Mekelle [21] and Jimma [20] revealed that respondents who had job stress were 2, 4.7 and 3.2 times more likely to develop WMSDs than those who had no job stress respectively. Another study done in Gorgan, Iran shows the average stress scores were significantly higher among those with MSDs [59]. Similar study done in Jimma discovered that computer users who were under stress had 3.2 times more risk of acquiring WRMSDs than those who were not [20]. Work stress appears as a mediating factor between the musculoskeletal pathology and the psy-chosocial factors [60]. There is no clear-cut understanding of how workplace psychosocial stress factors link to MSDs. However, it is well recognized that high mental and psychological stress may increase muscle tension and decrease micropauses in muscle activity. Due to low threshold motor units' constant firing, which is activated by both low-level physical loading and mental loading, this could cause muscle fatigue even under low loads [[20], [21], [22],^61^]. The nervous system and endocrine system are frequently the two areas of the human body where stress results in alterations. As a consequence, the human body's internal environment is continuously changing, and the body's adaptive mechanisms are constantly working to make adjustments [22,23]. The central nervous system's reaction to stress may amplify painful sensations, which would increase the prevalence of MSDs [62]. Psychosocial factors, like the support of co-workers, have an indirect effect on the symptoms and can attenuate the levels of psychosocial factors [63,64].

Rest was significantly associated with MSDs [21,41]. Break down the exposure to visual display terminals, enable a reduction in the muscle load brought on by poor ergonomics, and allow for muscle rest and recovery [65,66]. People who take breaks while working are more likely to relax their muscles and experience less discomfort [21]. However, a research conducted in Alexandria, Egypt, computer office workers who reported taking frequent breaks were 2.6 times more likely to experience neck complaints and 3.2 times more likely to experience arm or hand complaints than those who reported taking few or no breaks [9]. Employees who engaged in leisure activities at the end of the day felt less worn out than those who engaged in work or social activities during breaks [67]. Having greater levels of autonomy is not always associated with greater levels of beneficial relaxation activities. In other words, break time may provide little chance for rest and more chance for tasks that increase exhaustion and cause complaints [9].

Physical exercise was significantly associated with WMSDs [8,21]. A study conducted in south Gondar and Mekelle reported that participants who had never engaged in regular physical exercise had a 6- and 2.9-times higher chance of developing WMSDs than those who had, respectively [8,21]. Previous studies demonstrated physical activity programs at work reduce occupational stress and musculoskeletal pain. Worksite physical activity programs improve occupational health and positively affect the relief of musculoskeletal pain and perceived difficulty in performing task [64,68]. Exercise has a protective effect against the onset of musculoskeletal discomfort and injury. Physical activity for 20 min three times a week helps ease musculoskeletal discomfort in various body regions, including the shoulder, neck, and lumbar spine [4,69,70]. The participants who attended more weekly exercise sessions were 74 % more likely to report psychophysiological well-being, 30 % less likely to have difficulties in performing tasks, and 87 % more likely to perceive improved interpersonal relations [68]. A randomized controlled trial study found exercising at work place improved mood and performance, leading to better concentration, problem solving, a clearer mind, re-energization, work-based relationships and heightened resilience to stress. Exercising also presented a chance to interact with other – often less well known – colleagues. It offered an active break from the demands of the office, where participants commented on the marked contrast with the sedentary nature of their work [71].

A study from Jimma, Ethiopia showed respondents who admitted to drinking alcohol were 3.44 times more likely to develop WMSDs than those who did not [20], and another study done in Punja, India, revealed that respondents who regularly drank alcohol were more likely to experience WMSDs than those who did not [24]. The plausible explanation may be related to alcohol's negative impact on the body's normal physiology and defense mechanisms. Additionally, drinking alcohol may affect the behavior of those who frequently forbid them from leading a healthy lifestyle [72].

According to a study conducted in South Gondar [8] and Mekelle [21], respondents who did not undergo ergonomics training had a 5.4- and 3.8-times higher chance of developing WMSDs than those who did, respectively. Untrained individuals may lack the essential skills and information required to implement practical precautions against occupational musculoskeletal disorders, and as a result, they may fail to follow established protocols and work practices [8]. Musculoskeletal discomfort was greatly reduced by combining ergonomic training and exercises (from 10.5 to 52.6 % in various body locations) [38].

The likelihood of developing WMSDS was significantly associated with educational level, and those who had a low educational status were 4.2 times more likely to develop WMSDs than those who had a master's degree [21]. The probable explanation could be that those with lower educational levels may lack basic ergonomic knowledge and skills in the workplace [21].

Smokers were more affected by MSDs than nonsmokers [6,24,38]. Cigarette smoking has deleterious effects on the musculo-skeletal system and worsens the prognosis of several orthopaedic disorders. It decreases bone mineral content and increases the incidence of fractures. Due to the direct toxic effects on osteoblasts/osteoclasts activity of nicotine and its indirect actions on sex and adrenocortical hormones, vitamin D, intestinal calcium absorption, vessels, and oxygen supply [73].

Studies done in Saudi Arabia (Dhaka) [4], Egypt (Alexandria), Ghana (Kumasi) [23] and China [41] discovered that using computer for extended periods of time significantly increased the chance of developing MSDs.

## 6 Limitation

Since the present study is a systematic review, hence statistical analysis was not performed, as a result, the aggregated results were not presented.

Sample size variability was observed; therefore, future scholars should be given into consideration.

Publication year, journal impact factor and sample size were not considered during article selection, therefore the coming scholars should be considered it.

In this review, the regional prevalence of WMSDs was not investigated.

## 7 Conclusion

The prevalence of WMSDs among computer user bankers, and office workers was very high. Therefore, the government and other stakeholders should take action to limit the burden of MSDs on the health of workers.

The majority of articles reported over 50 % of computer users who worked as bankers or office workers experienced WMSDs.

Most of the articles confirmed that, the lower back, neck, upper back, and shoulder were the most affected body parts, and the elbow, hip/thigh, knee, wrist/hand, and ankle/feet were the least affected.

Being female is the most frequently reported risk factor, followed by working in awkward posture, being older, having no or less brake time, and lack of ergonomics training.

## 8 Recommendation

Reducing prolonged use of computers, providing sufficient break time during work, doing physical exercise and working in the right posture will reduce the magnitude of MSD among computer users.

Risk factors associated with MSD were identified; therefore, an interventional study should be done.

To present the aggregated result for readers and scholars, meta-analysis should be done.

The regional prevalence of WMSDs should be conducted.

## Funding statement

No funds were obtained for this particular study.

## Data availability statement

All data are available in the manuscript.

## CRediT authorship contribution statement

**Biruk Demissie:** Writing – review & editing, Writing – original draft, Conceptualization. **Eniyew Tegegne Bayih:** Writing – review & editing, Supervision, Investigation. **Alelign Alemu Demmelash:** Writing – review & editing, Supervision.

## Declaration of competing interest

The authors declare that they have no known competing financial interests or personal relationships that could have appeared to influence the work reported in this paper.
