# Supplementary material for: A systematic review of work-related musculoskeletal disorders and risk factors among computer users
Source: Heliyon. 2024 Jan 22;10(3):e25075. doi: 10.1016/j.heliyon.2024.e25075 (PMC10840111; doi:10.1016/j.heliyon.2024.e25075)
Supplement: Multimedia component 1 [file mmc1.docx]

**A systematic review of work-related musculoskeletal disorders and risk factors among computer users**

Biruk Demissie^a^, Eniyew Tegegne Bayih^b^ and Alelign Alemu Demmelash^c^

^a^ Department of Environmental health, College of health science, Debre Tabor university, Debre Tabor, Ethiopia

^b^Department of Environmental health, College of health science, Debre Markos University, Debre Markos, Ethiopia

^c^Department of Environmental Health and Hygiene, Bonn Universiy Hospital, Bonn, Germany

Correspondence author: Biruk Demissie

Email: [brookmelse2022@gmail.com](mailto:brookmelse2022@gmail.com)

ETB: eniyewtegegne@gmail.com

AAD: alemualelign@gmail.com

**Table 1 PRISMA 2020 for Abstracts Checklist**

| **Section and Topic** | **Item #** | **Checklist item** | **Reported (Yes/No)** |
| --- | --- | --- | --- |
| **TITLE** | | |  |
| Title | 1 | Identify the report as a systematic review. | Yes |
| **BACKGROUND** | | |  |
| Objectives | 2 | Provide an explicit statement of the main objective(s) or question(s) the review addresses. | Yes |
| **METHODS** | | |  |
| Eligibility criteria | 3 | Specify the inclusion and exclusion criteria for the review. | Yes |
| Information sources | 4 | Specify the information sources (e.g. databases, registers) used to identify studies and the date when each was last searched. | Yes |
| Risk of bias | 5 | Specify the methods used to assess risk of bias in the included studies. | Yes |
| Synthesis of results | 6 | Specify the methods used to present and synthesis results. |  |
| **RESULTS** | | |  |
| Included studies | 7 | Give the total number of included studies and participants and summarize relevant characteristics of studies. | Yes |
| Synthesis of results | 8 | Present results for main outcomes, preferably indicating the number of included studies and participants for each. If meta-analysis was done, report the summary estimate and confidence/credible interval. If comparing groups, indicate the direction of the effect (i.e. which group is favored). | Yes |
| **DISCUSSION** | | |  |
| Limitations of evidence | 9 | Provide a brief summary of the limitations of the evidence included in the review (e.g. study risk of bias, inconsistency and imprecision). | Yes |
| Interpretation | 10 | Provide a general interpretation of the results and important implications. | Yes |
| **OTHER** | | |  |
| Funding | 11 | Specify the primary source of funding for the review. | Not applicable |
| Registration | 12 | Provide the register name and registration number. | Not registered |

*From:* Page MJ, McKenzie JE, Bossuyt PM, Boutron I, Hoffmann TC, Mulrow CD, et al. The PRISMA 2020 statement: an updated guideline for reporting systematic reviews. BMJ 2021;372:n71. doi: 10.1136/bmj.n71

For more information, visit: <http://www.prisma-statement.org/>
