# Supplementary material for: A systematic review of work-related musculoskeletal disorders and risk factors among computer users
Source: Heliyon. 2024 Jan 22;10(3):e25075. doi: 10.1016/j.heliyon.2024.e25075 (PMC10840111; doi:10.1016/j.heliyon.2024.e25075)
Supplement: Multimedia component 2 [file mmc2.docx]

**A systematic review of work-related musculoskeletal disorders and risk factors among computer users**

Biruk Demissie^a^ , Eniyew Tegegne Bayih^b^ and Alelign Alemu Demmelash^c^

^a^ Department of Environmental health, College of health science, Debre Tabor university, Debre Tabor, Ethiopia

^b^Department of Environmental health, College of health science, Debre Markos University, Debre Markos, Ethiopia

^c^Department of Environmental Health and Hygiene, Bonn Universiy Hospital, Bonn, Germany

Correspondence Author: Biruk Demissie

Email: [brookmelse2022@gmail.com](mailto:brookmelse2022@gmail.com)

ETB: eniyewtegegne@gmail.com

AAD: alemualelign@gmail.com

Table 1 Quality assessment of the included studies using the Joanna Briggs Institute (JBI) quality appraisal criteria

| **For cross-sectional studies** | | | | | | | | | | | |
| --- | --- | --- | --- | --- | --- | --- | --- | --- | --- | --- | --- |
| S/N | **Author** | **Criteria** | | | | | | | | Score | Overall quality |
|  |  | Clearly defined inclusion criteria | Describing the study settings participants | Valid &reliable exposure measurement | Objective &standard criteria for measurement | Identified confounder | Strategies to deal with confounder | Valid & reliable outcome measurement | Appropriate statistical analysis |  |  |
| 1 | Dagne et al | Y | Y | Y | Y | N | Y | Y | Y | 7 | Low risk |
| 2 | Dixit et al | Y | N | Y | Y | N | Y | Y | Y | 6 | Low risk |
| 3 | Amin et al | N | N | Y | Y | N | Y | Y | Y | 5 | Low risk |
| 4 | Noha S. Elshaer | Y | N | Y | Y | N | Y | Y | Y | 6 | Low risk |
| 5 | Darvishi et al | Y | Y | Y | Y | N | Y | Y | Y | 7 | Low risk |
| 6 | Pate et al | Y | Y | Y | Y | N | Y | Y | Y | 7 | Low risk |
| 7 | Abdullah S. & Abdullah M | Y | Y | Y | Y | N | Y | Y | Y | 7 | Low risk |
| 8 | Jubilant K & Godfred K | Y | Y | Y | Y | N | Y | Y | Y | 7 | Low risk |
| 9 | Khan et al | Y | Y | Y | Y | N | N | Y | Y | 6 | Low risk |
| 10 | I. T. S. Yu & T. W. Wong | N | Y | Y | Y | N | Y | Y | Y | 6 | Low risk |
| 11 | Dixit et al | Y | Y | Y | Y | N | Y | Y | Y | 7 | Low risk |
| 12 | Sulaiman er al | Y | Y | Y | Y | N | N | Y | Y | 6 | Low risk |
| 13 | Etana et al | Y | Y | Y | Y | N | Y | Y | Y | 7 | Low risk |
| 14 | Akrouf et al | N | N | Y | Y | N | Y | Y | Y | 5 | Low risk |
| 15 | Demissie et al | Y | Y | Y | Y | N | Y | Y | Y | 7 | Low risk |
| 16 | Kibret et al | Y | Y | Y | Y | N | Y | Y | Y | 7 | Low risk |
| 17 | Maduagwu et al | Y | N | Y | Y | N | N | Y | Y | 5 | Low risk |
| 18 | Ranasinghe P et al | Y | Y | Y | Y | N | Y | Y | Y | 7 | Low risk |
| 19 | Shanshan Wu et al | Y | Y | Y | Y | N | Y | Y | Y | 7 | Low risk |
| 20 | Oha K et al | Y | N | Y | Y | N | Y | Y | Y | 6 | Low risk |
| 21 | Latha S et al | Y | N | Y | Y | N | N | Y | Y | 5 | Low risk |
| 22 | Navidi F et al | Y | N | Y | Y | N | Y | Y | Y | 6 | Low risk |
| 23 | Aziz A & Azmi N | N | Y | Y | Y | N | N | Y | Y | 5 | Low risk |
| 24 | Habibi E et al | Y | N | Y | Y | N | Y | Y | Y | 6 | Low risk |
| 25 | Noraziera Z & Norzaida A | Y | N | Y | Y | N | Y | Y | Y | 6 | Low risk |

*Note: Y, yes; N, No*

Table 2 supplemental file on Risk of bias assessment of the included studies

| **S/N** | **Author [Year]** | **Criteria** | | | | | | | | | | **Scores** | **Overall risk of bias** |
| --- | --- | --- | --- | --- | --- | --- | --- | --- | --- | --- | --- | --- | --- |
|  |  | **External validity** | | | | **Internal validity** | | | | | |  |  |
|  |  | **Q1** | **Q2** | **Q3** | **Q4** | **Q5** | **Q6** | **Q7** | **Q8** | **Q9** | **Q10** |  |  |
|  | Dagne et al | Y | Y | Y | Y | Y | Y | Y | Y | Y | Y | 10 | Low risk |
|  | Dixit et al | Y | Y | Y | Y | Y | Y | Y | Y | Y | Y | 10 | Low risk |
|  | Amin et al | Y | Y | N | Y | Y | N | Y | Y | Y | Y | 8 | Low risk |
|  | Noha S. Elshaer | Y | Y | N | Y | Y | Y | Y | Y | Y | Y | 9 | Low risk |
|  | Darvishi et al | Y | Y | Y | Y | Y | Y | Y | Y | Y | Y | 10 | Low risk |
|  | Pate et al | Y | Y | N | Y | Y | N | Y | Y | Y | Y | 8 | Low risk |
|  | Abdullah S. & Abdullah M | Y | Y | N | N | Y | N | Y | Y | Y | Y | 7 | Low risk |
|  | Jubilant K & Godfred K | Y | Y | N | Y | Y | N | Y | Y | Y | Y | 8 | Low risk |
|  | Khan et al | N | Y | Y | Y | Y | N | Y | Y | Y | Y | 8 | Low risk |
|  | I. T. S. Yu & T. W. Wong | N | N | N | Y | Y | N | Y | Y | Y | Y | 6 | Low risk |
|  | Dixit et al | N | N | N | Y | Y | N | Y | Y | Y | Y | 6 | Low risk |
|  | Sulaiman er al | Y | Y | Y | Y | Y | N | Y | Y | Y | Y | 9 | Low risk |
|  | Etana et al | Y | Y | Y | Y | Y | Y | Y | Y | Y | Y | 10 | Low risk |
|  | Akrouf et al | Y | N | N | Y | Y | N | Y | Y | Y | Y | 7 | Low risk |
|  | Demissie et al | Y | Y | Y | Y | Y | Y | Y | Y | Y | Y | 10 | Low risk |
|  | Kibret et al | Y | Y | Y | Y | Y | Y | Y | Y | Y | Y | 10 | Low risk |
|  | Maduagwu et al | Y | N | N | Y | Y | N | Y | Y | Y | Y | 7 | Low risk |
|  | Ranasinghe P et al | Y | N | N | Y | Y | N | Y | Y | Y | Y | 7 | Low risk |
|  | Shanshan Wu et al | Y | N | N | Y | Y | N | Y | Y | Y | Y | 7 | Low risk |
|  | Oha K et al | Y | Y | Y | Y | Y | N | Y | Y | Y | Y | 9 | Low risk |
|  | Latha S et al | Y | Y | N | Y | Y | N | Y | Y | Y | Y | 8 | Low risk |
|  | Navidi F et al | N | Y | N | N | Y | N | Y | Y | Y | Y | 6 | Low risk |
|  | Aziz A & Azmi N | N | Y | N | N | Y | N | Y | Y | Y | Y | 6 | Low risk |
|  | Habibi E et al | N | Y | N | N | Y | N | Y | Y | Y | Y | 6 | Low risk |
|  | Noraziera Z & Norzaida A | N | Y | N | N | Y | N | Y | Y | Y | Y | 6 | Low risk |

Note: Y, Yes; N, No; Q1, Representatives of the target population; Q2, Representativeness of the sampling frame; Q3, Random sampling or census; Q4, Minimal response bias; Q5, Data were collected directly; Q6, Acceptable case definition used in the study; Q7, Valid and reliable measurement; Q8, The same mode of data collection for all study subject; Q9, Appropriate length of prevalence period for parameter of interest and Q10, Appropriate numerators and denominators of interest.
